# Implementation of a workplace healthy lifestyle education intervention for metabolic syndrome risk reduction in Ethiopia: a mixed-methods process evaluation

**DOI:** 10.3389/fpubh.2026.1893222

**Published:** 2026-10-07

**Authors:** Sitotaw Kerie Bogale, Haribondhu Sarma, Uday Narayan Yadav, Matthew Kelly

**Affiliations:** 1 National Centre for Epidemiology and Population Health, The Australian National University, Canberra, ACT, Australia; 2 College of Medicine and Health Sciences, Bahir Dar University, Bahir Dar, Ethiopia; 3 International Centre for Future Health Systems, University of New South Wales, Sydney, NSW, Australia; 4 The Poche Centre for Indigenous Health, Faculty of Medicine and Health, The University of Sydney, Sydney, NSW, Australia

**Keywords:** Ethiopia, fidelity, implementation, lifestyle intervention, metabolic syndrome, office workers, risk factors, workplace

## Abstract

**Background:**

The prevalence of metabolic syndrome among office workers is markedly high, highlighting an urgent need for effective workplace interventions. In response to this need, a theory-informed healthy lifestyle education intervention was developed and implemented among office workers in Ethiopia, resulting in significant reductions in key metabolic risk factors and improvements in health-related behaviours. However, understanding the implementation process, how the intervention worked in practice, under what conditions, and why is critical to inform scale-up, adaptation, and sustainability. This study aimed to evaluate the implementation process of a nine-month workplace-based healthy lifestyle education intervention, using the Programme Integrity Framework to assess implementation fidelity and the Consolidated Framework for Implementation Research to identify the barriers and facilitators influencing implementation.

**Methods:**

A convergent parallel mixed-methods process evaluation was conducted to assess the implementation process of a 9 month workplace intervention aimed to reduce risk for metabolic syndrome and improve knowledge, attitudes, and healthy lifestyle practices. A total of 226 employees were screened based on waist circumference and blood pressure and allocated to intervention and control groups using cluster randomisation. The intervention group received a personalised lifestyle education programme delivered by trained health promotion experts, while the control group received general health advice on non-communicable disease prevention. Implementation fidelity was assessed across the five domains of the Programme Integrity Framework: adherence, exposure, quality of delivery, participant responsiveness, and programme differentiation, using educator self-reports and direct observations conducted by the principal investigator. Contextual factors influencing implementation were examined using the five domains of the Consolidated Framework for Implementation Research (CFIR): intervention characteristics, outer setting, inner setting, characteristics of individuals, and process. These were assessed through participant self-reports, field notes from review meetings conducted at the third and sixth months, and open-ended questionnaire responses collected from 13 participants.

**Results:**

Implementation fidelity was high across adherence, exposure, quality of delivery, and participant responsiveness domains, indicating that the intervention was delivered as intended and well-received by participants. However, CFIR-informed contextual analysis identified several barriers to effective implementation, including unsupportive organisational cultures, limited leadership support, weak external policy backing, resource constraints, and community norms that were not always aligned with healthy lifestyle change.

**Conclusion:**

This study shows that a theory-informed lifestyle education intervention can be delivered with high fidelity and strong engagement among office workers, supporting workplaces as a promising NCD prevention setting. Sustainable scale-up will likely depend on task-sharing with trained non-specialist personnel and embedding workplace health promotion within organisational and policy systems. Future research should work on policy integration, scalability, long-term sustainability, equity, and cost-effectiveness.

**Clinical trial registration:**

https://www.anzctr.org.au/Trial/Registration/TrialReview.aspx?id=385666&isClinicalTrial=False, identifier ACTRN12623000409673p.

## Introduction

Metabolic syndrome (MetS), characterised by a clustering of high blood pressure, elevated blood glucose, central obesity, and dyslipidaemia, substantially increases the risk of non-communicable diseases (NCDs), particularly cardiovascular disease (CVD) and type 2 diabetes mellitus (T2DM) (1, 2).

Globally, MetS affects well over one billion people and has become a major public health challenge (3). Its burden is particularly high in low- and middle-income countries (LMICs), where prevalence varies across regions and populations. Pooled estimates indicate a prevalence of approximately 30% among adults in India (4, 5) and 32.4% in Africa (6). This growing burden has been linked to rapid lifestyle transitions, including shifts from traditional to more sedentary patterns of living, poor dietary habits, insufficient physical activity, and increased consumption of alcohol and tobacco (3).

Office workers are especially vulnerable to MetS because of prolonged sitting, limited physical activity during working hours, work-related stress, and inadequate workplace support for healthy behaviours (7–9). Studies among office workers in Iran and Indonesia have reported high MetS prevalence, ranging from 36 to 38.7% (10, 11).

In Ethiopia, MetS prevalence among office workers has been estimated at 27.6% using the International Diabetes Federation criteria (12). Individual knowledge, attitudes, and awareness of MetS, CVDs, and behavioural risk factors also play an important role in shaping lifestyle behaviours and may mediate the development of MetS risk (13). These findings emphasise the necessity of contextually appropriate workplace interventions to reduce MetS risk and promote healthier behaviours among office workers in Ethiopia.

Bahir Dar, the second largest city in Ethiopia, provides an important setting for such interventions. The city has a growing burden of MetS and CVD risk factors, including low fruit and vegetable intake, insufficient physical activity, limited awareness of cardiovascular risk factors, and work-related stress among adult office workers (14–16). Bank employees may be particularly at risk because their work is predominantly sedentary and often involves prolonged sitting, psychosocial stress, and limited opportunities for physical activity during working hours (15, 16). Our previous qualitative study among office workers in Bahir Dar City, currently under review elsewhere, identified several barriers to healthy lifestyle adoption, including limited access to healthy foods and sports facilities, safety concerns around outdoor physical activity, widespread availability and social acceptance of alcohol consumption, and cultural influences on diet and lifestyle. Structured workplace wellness programmes also remain limited in this setting. Broader contextual factors, including underdeveloped initiatives to improve access to healthy foods, such as urban agriculture, and the persistence of unhealthy dietary preferences and alcohol use during social gatherings, may further constrain behaviour change (17). These challenges also compounded by the ongoing conflict in the region by disrupting mobility, livelihoods, services, and access to health-promoting resources. Together, these individual, workplace, community, and wider contextual factors underscore the need for targeted workplace-based health education interventions to reduce MetS and CVD risk among bank employees in Bahir Dar City.

In response to this need, a theory-informed healthy lifestyle education intervention was developed and implemented among office workers in Ethiopia. The intervention aimed to promote healthy dietary practices, increase physical activity, reduce harmful alcohol consumption, support smoking cessation, and improve stress management. It was informed by contemporary behaviour change approaches that emphasise theory-based design, contextual adaptation, personalised recommendations, stakeholder engagement, and appropriate delivery strategies. Evidence from other settings suggests that these principles can strengthen the design, implementation, and local relevance of chronic disease prevention interventions (18). The intervention was effective in reducing MetS risk factors, improving awareness of MetS, CVDs, and related risk factors, and promoting healthy lifestyle behaviours (19).

However, evidence of effectiveness alone is insufficient to support sustainability, replication, and scale-up. Understanding how an intervention was implemented, whether it was delivered as intended, and what factors influenced implementation is critical for interpreting trial outcomes and informing future adaptation. Therefore, this study aimed to assess intervention fidelity across the five domains of the programme integrity framework and to identify implementation barriers and facilitators across the five domains of the Consolidated Framework for Implementation Research.

### Conceptual frameworks

This process evaluation study was meticulously guided by established frameworks, such as the Programme Integrity Framework (PIF) (20) and the original Consolidated Framework for Implementation Research (CFIR), which includes five domains and 39 constructs (21). These frameworks have been applied in various settings and have proven helpful in evaluating the implementation process (22, 23). The PIF was used to assess implementation fidelity across five dimensions: adherence, exposure or dose, quality of delivery, participant responsiveness, and programme differentiation. The CFIR guided the assessment of contextual factors influencing implementation across five domains: intervention characteristics, outer setting, inner setting, characteristics of individuals involved, and process factors.

## Methods and materials

### Design

This process evaluation study, rooted within a broader implementation research project, used a convergent parallel mixed-methods design. Quantitative and qualitative data were collected during the same implementation period, analysed separately, and then integrated at the interpretation stage. Intervention fidelity and contextual determinants influencing implementation of the workplace healthy lifestyle education intervention were assessed. The intervention was designed and implemented to reduce MetS risk factors and to examine changes in participants’ knowledge and attitudes related to MetS, CVDs and associated risk factors, as well as healthy lifestyle behaviours. The effectiveness of the intervention was evaluated using a cluster-randomised controlled trial (RCT) design among Bahir Dar City bank employees (19). Because Bahir Dar City is divided into two geographical clusters by the Abay River, banks in one area were allocated to the intervention arm and those in the other area to the control arm. Due to the nature of the intervention, participants could not be blinded to group allocation, but they were unaware of their assigned arm, while data collectors, laboratory personnel, and the intervention provider remained blinded.

The present process evaluation specifically focuses on assessing intervention fidelity and elucidating the multilevel contextual factors that shaped the implementation process. This study was conducted following the Declaration of Helsinki. The ethical approvals were obtained from the Human Ethics Committee at the Australian National University and the Institutional Review Board at Bahir Dar University College of Medicine and Health Sciences (with unique protocol IDs 2022/845 and 792/2023, respectively).

### Study setting

This study was conducted in Bahir Dar City, the capital of the Amhara Region, located 565 kilometres northwest of Addis Ababa, Ethiopia. Bahir Dar was selected as the study setting because of its reported high burden of metabolic syndrome and cardiovascular disease, as well as the presence of related behavioural and occupational risk factors, including low fruit and vegetable intake, insufficient physical activity, limited awareness of cardiovascular risk factors, and work-related stress (14–16). The city has 210 bank branches employing approximately 2,310 individuals, providing a large and occupationally stable population suitable for a workplace-based intervention.

### Participants and recruitment

Between 29 September 2023 and 1 January 2024, 112 bank branches were approached, including 36 government-owned branches and the remainder private banks, and participants were recruited from both sectors. Recruitment was conducted through obtaining official letters from the local health department office of the Ministry of Health within the city administration to district bank headquarters, followed by communication with branch managers. Potential participants were informed in person about the study objectives, procedures, eligibility criteria, risks, benefits, and ethical considerations, and written informed consent was obtained before screening. A total of 1,574 employees were approached, 864 underwent workplace screening for blood pressure and central obesity, and 607 were excluded because they had normal readings. Ultimately, 226 employees who met the eligibility criteria were enrolled and assigned to groups according to their workplace. Employees were eligible if they had central obesity and/or elevated blood pressure. Central obesity was defined as a waist circumference >102 cm in men and >88 cm in women (24), measured at the midpoint between the lower margin of the last palpable rib and the top of the iliac crest, or at the point of maximum abdominal extension. Elevated blood pressure was defined as blood pressure ≥130/85 mmHg (24, 25). Blood pressure was measured three times using an appropriately sized sphygmomanometer after a 5-min rest, with 5-min intervals between measurements, and the mean systolic and diastolic values were used for classification. No prior confirmed diagnosis was required for enrolment. Exclusion criteria included dietary restrictions, contraindications to physical activity due to musculoskeletal, neurological, vascular, pulmonary, or cardiac conditions, pregnancy or planned pregnancy, severe psychiatric disorders, significant cognitive impairment, and inability to commit to the full programme.

For this process evaluation study, 13 participants were selected from the 105 intervention completers. These participants were purposively selected to ensure variation in age, sex, job role, and bank type. The sample included participants aged 32–58 years, with representation from both sexes, a range of occupational roles (customer service staff, senior officers, and branch managers), and both private and government banks (Table 1). This purposive approach was intended to capture a range of perspectives and to reflect the diversity of the full intervention group rather than to achieve statistical representativeness. Additionally, self-reported data on barriers and facilitators to implementing intervention recommendations were collected from all 105 participants using a standardised template. All participants provided written informed consent before participating. The details of requirement and allocation are presented in Figure 1.

**Table 1:** Characteristics of participants completing the open-ended CFIR-based component (*n* = 13).

Participant ID	Age in year	Sex	Job role	Bank type
088	36	Male	Branch manager	Private
082	38	Male	Senior officer	Government
093	42	Male	Senior officer	Government
100	56	Male	Senior officer	Private
053	44	Male	Senior officer	Government
102	58	Male	Head office manager	Private
066	46	Female	Senior officer	Private
014	58	Male	Branch manager	Government
064	45	Male	Senior officer	Private
005	34	Female	Senior officer	Government
080	32	Female	Customer service	Private
009	54	Female	Branch manager	Government
015	43	Male	Branch manager	Government

Source: Authors’ own elaboration.

**Figure 1:**
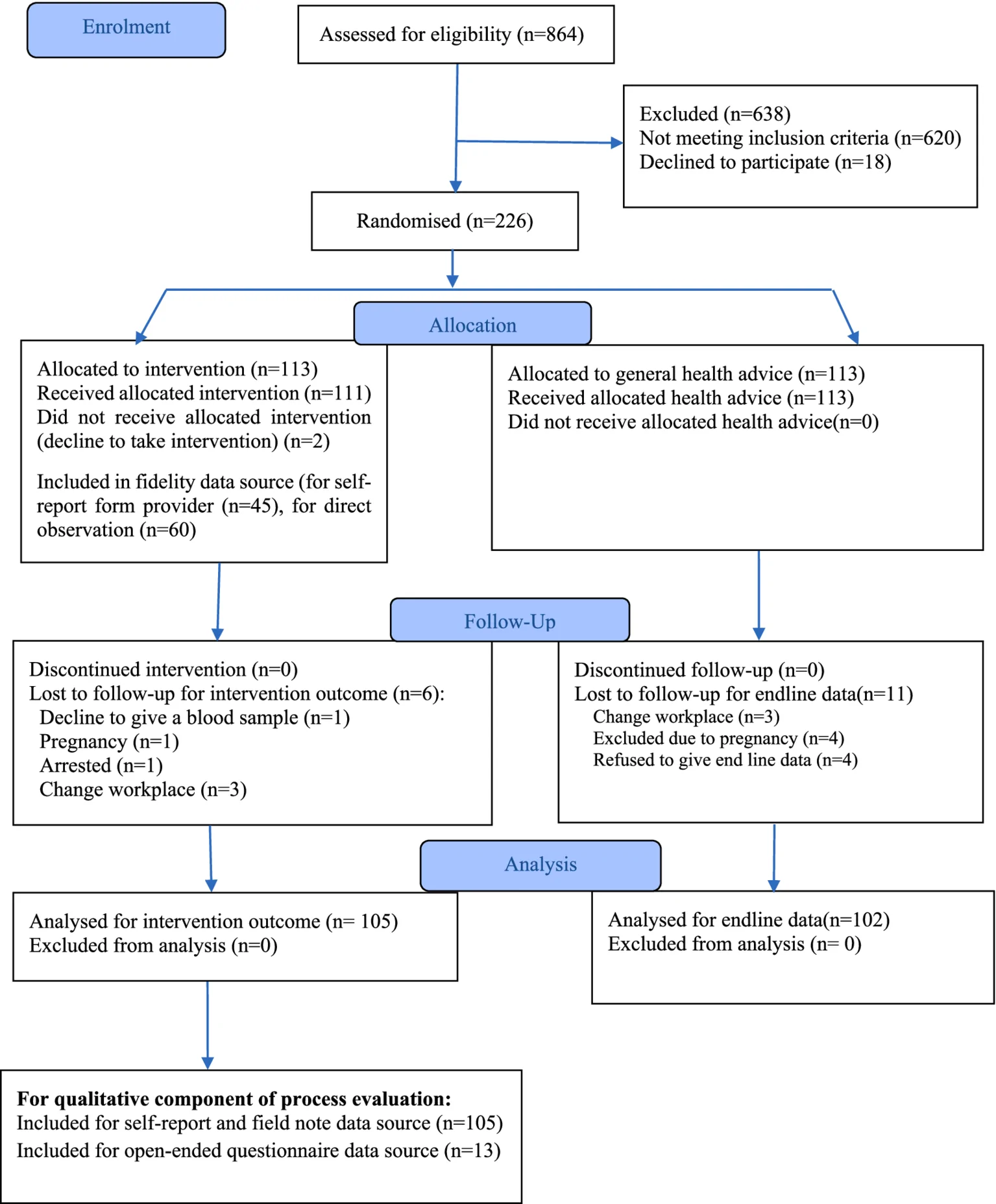
Trial flow diagram for the implementation process evaluation of intervention.

### Intervention

#### Intervention description

The intervention was a one-to-one workplace-based healthy lifestyle education programme designed to reduce the risk of MetS among bank employees. The intervention was developed in collaboration with experts in nutrition, psychology, and sports science. Its content focused on MetS, its risk factors, prevention strategies, and lifestyle modification, including healthy eating, regular physical activity, alcohol reduction, smoking cessation, and stress management.

#### Development of the intervention

The educational content was prepared in collaboration with subject-matter experts in nutrition, psychology, and sports science. The intervention materials included a printed PowerPoint-based education package, an action tracking template, and structured reminder text messages. The materials were designed to provide consistent information across participants while allowing individualisation according to each participant’s lifestyle risks and needs.

#### Training of intervention providers

The intervention was delivered by three health promotion experts. Before implementation, the providers received a half-day training session led by the principal investigator. The training covered the aim and rationale of the intervention, the intervention protocol, key educational content, use of the printed PowerPoint presentation, use of the activity tracking template, counselling approaches, and methods for responding to participant questions and concerns. The training was intended to standardise intervention delivery and ensure consistency across providers.

#### Intervention setting and mode of delivery

The intervention was delivered face to face in the participants’ workplaces, specifically at bank branches in Bahir Dar City, Ethiopia. The intervention was originally planned to be delivered in a group format. However, because a state of emergency was declared in the region due to civil conflict, public gatherings were prohibited. Therefore, the delivery mode was modified from group-based education to one-to-one education. No changes were made to the core educational content.

#### Intervention procedures

Each participant in the intervention group received an individual face-to-face education session lasting approximately 1 h and 45 min to 2 h. During the session, the health promotion expert explained metabolic syndrome, its risk factors, and prevention strategies. The educator then discussed key lifestyle behaviours, including dietary habits, physical activity, alcohol consumption, smoking, and stress management.

The education session followed the printed PowerPoint material to ensure consistency. However, counselling was tailored to the participant’s individual lifestyle profile and needs. Participants were encouraged to identify personal lifestyle risks and to select feasible behaviour-change goals. At the end of the session, each participant received the printed educational material and an activity tracking template.

Participants were instructed to record their lifestyle-related activities using the provided template during the implementation period. The template was used to help participants monitor behaviours such as physical activity, dietary changes, alcohol reduction, smoking cessation efforts, and stress management practices.

#### Reinforcement and follow-up support

To reinforce the intervention content, participants received reminder text messages every 2 weeks throughout the intervention implementation period. The messages summarised key concepts from the education session, encouraged participants to continue implementing lifestyle changes, and reminded them to use the activity tracking template. Participants were also encouraged to ask questions and seek advice from the intervention providers during the implementation period.

In addition, the principal investigator conducted review meetings at the third and sixth months after the initial education session. Each review meeting lasted approximately 45 min to 1 h. These meetings were used to revisit the main intervention messages, discuss participants’ progress, address challenges encountered during implementation, and respond to participants’ questions or concerns.

#### Timing and dose of the intervention

The initial one-to-one education sessions were delivered between 29 September 2023 and 1 January 2024. Each participant received one individual education session lasting 1 h and 45 min to 2 h. Follow-up reinforcement consisted of text messages sent every 2 weeks during the implementation period and review meetings conducted at the third and sixth months. Each review meeting lasted 45 min to 1 h.

#### Tailoring

Although the intervention followed a standardised educational package, the counselling component was individualised. Tailoring was based on participants’ lifestyle behaviours, perceived barriers, and personal circumstances. For example, discussions on diet, physical activity, alcohol intake, smoking cessation, and stress management were adapted according to the participants’ current practices and readiness to change.

#### Fidelity monitoring

Intervention fidelity was assessed using the Programme Integrity Framework, which includes adherence, exposure or dose, quality of delivery, participant responsiveness, and programme differentiation. Fidelity was assessed through intervention provider reports and direct observation by the principal investigator using a structured checklist.

#### Control group

Participants in the control group received individual general health advice delivered by a BSc nurse in accordance with the national non-communicable disease management guideline (26). The control group did not receive the structured healthy lifestyle education package, printed PowerPoint material, activity tracking template, biweekly reminder text messages, or scheduled review meetings provided to the intervention group (see Table 2).

**Table 2:** Summary of the workplace healthy lifestyle education intervention for metabolic syndrome risk reduction.

Item	Description
Brief name	One-to-one workplace healthy lifestyle education intervention for metabolic syndrome risk reduction
Aim	To reduce MetS risk by promoting lifestyle changes related to healthy eating, physical activity, alcohol reduction, smoking cessation, and stress management
Theoretical/content basis	Developed with experts in nutrition, psychology, and sports science; focused on MetS, risk factors, prevention strategies, and lifestyle modification
Materials	Printed PowerPoint-based education package, activity tracking template, and reminder text messages sent every 2 weeks
Intervention providers	Three trained health promotion experts
Provider training	Providers received half-day training before implementation. Training covered the intervention rationale, protocol, key content areas, use of intervention materials, individualised counselling, and management of participant questions and concerns
Setting	Workplace setting, bank branches in Bahir Dar City, Ethiopia
Mode of delivery	Face-to-face, one-to-one education sessions, supported by printed materials, activity tracking, text-message reminders, and review meetings
Initial session	Each participant received one individual education session lasting approximately 1 h and 45 min to 2 h
Core content	Metabolic syndrome definition and consequences; risk factors; prevention strategies; healthy eating; physical activity; alcohol reduction; smoking cessation; and stress management
Participant activities	Participants were encouraged to set personal lifestyle goals, use the activity tracking template, record lifestyle-related activities, ask questions, and seek advice during the implementation period
Reinforcement	Reminder text messages were sent every 2 weeks to reinforce key messages and encourage continued lifestyle modification
Follow-up/review	Review meetings were conducted by the principal investigator at the third and sixth months. Each meeting lasted 45 min to 1 h and focused on reviewing key messages, addressing concerns, and supporting continued behaviour change
Timing	Initial education sessions were delivered from 29 September 2023 to 1 January 2024
Tailoring	Counselling was individualised according to participants’ lifestyle behaviours, needs, barriers, and personal circumstances
Modification	Delivery changed from group-based to one-to-one education because a state of emergency related to civil conflict prohibited public gatherings. The core intervention content remained unchanged
Fidelity assessment	Fidelity was assessed using the Programme Integrity Framework: adherence, exposure/dose, quality of delivery, participant responsiveness, and programme differentiation. Data were collected from provider reports and direct observation by the principal investigator using a checklist
Actual fidelity	Adherence: 95%; exposure/dose: 85.71%; quality of delivery: 91.67%; participant responsiveness: 93.75%; programme differentiation: 58.33%
Control condition	Individual general health advice delivered by a BSc nurse according to national non-communicable disease management guidelines

Source: Authors’ own elaboration.

### Data collection for process evaluation

#### Quantitative data

The fidelity checklist was developed by the principal investigator based on the Programme Integrity Framework and was reviewed by experts in public health and implementation research to ensure content validity. Data about fidelity were collected from October 1, 2023, to January 1, 2024, through reports from one of the education providers who taught 45 participants and direct observations by the principal investigator. Three health promotion experts delivered the one to one education intervention to 111 participants. Fidelity assessment focused on one purposively selected provider who delivered 45 sessions (40.5%), representing the largest portion of intervention delivery. Fidelity data were not collected from the remaining providers, as the assessment prioritised in-depth evaluation of a single provider rather than sampling across all providers.

A total of 60 sessions (54.1%) were directly observed by the principal investigator across all providers, sampled across early, mid, and late delivery phases. The principal investigator completed observational fidelity checklists, while the provider completed corresponding self-reports. Given that the selected provider delivered a substantial proportion of sessions and observations covered over half of these across all phases, fidelity findings are considered reasonably representative; however, the absence of data from other providers may limit generalisability.

*Adherence* was evaluated with 5 items (out of 20), *exposure or dose* was assessed with 4 items (out of 14), *quality of delivery* was measured with 5 items (maximum score of 18), *participant responsiveness* was evaluated with 4 items (out of 16), and *programme differentiation* was assessed with three questions (out of 12) (Supplementary File 1: Fidelity assessment checklist).

#### Qualitative data

Qualitative data on contextual factors to implementation were collected using three sources: participant self-reports, field notes from review meetings conducted at the third and sixth months, and a 17-item open-ended questionnaire developed in Amharic language based on CFIR constructs. Self-reported data were obtained from all 105 intervention completers using a structured template developed by the research team. Participants recorded barriers and facilitators to implementing the recommended healthy lifestyle routine during the entire intervention period. These data captured ongoing, practice-oriented experiences.

In contrast, the open-ended questionnaire was administered to a purposive subsample of 13 participants at the end of the intervention. Participants were informed about the study objectives and were given 3 weeks to provide detailed written responses. The questionnaire was explicitly designed to elicit in-depth, theory-informed reflections aligned with CFIR domains, thereby generating richer and more analytically detailed data.

All qualitative data sources were analysed concurrently. Self-reports and field notes contributed to identifying recurrent, experience-based barriers and facilitators over time, while open-ended responses supported deeper interpretation and systematic mapping to CFIR constructs. The integration of these complementary data sources enabled triangulation and strengthened the robustness of the findings (Supplementary File 2: CFIR based open-ended questions).

#### Data management and analysis

For quantitative fidelity data, descriptive statistics, including percentage calculations, were used to assess fidelity levels based on self-reports from education providers and direct observations by the principal investigator. Raw scores were converted to percentages using the formula: Percentage = (obtained raw score ÷ maximum possible score) × 100. Fidelity levels were classified *a priori* as high (≥80%), moderate (60–79%), or low (<60%). These thresholds were selected as pragmatic benchmarks informed by commonly used fidelity-rating practices and applied consistently across the assessed intervention components. Because no universally accepted cut-offs exist for implementation fidelity, these categories should be interpreted as study-specific operational definitions rather than validated universal standards (27, 28).

Qualitative analysis followed a staged process of familiarisation, indexing, charting, organisation, and interpretation. Data were collected in Amharic and translated into English before analysis, with translation accuracy ensured through a bilingual researcher review process in which translated texts were cross-checked against the original data. All qualitative data (open-ended responses, self-reports, and field notes) were imported into NVivo version 14 and analysed using a hybrid approach combining inductive coding with deductive mapping to CFIR constructs.

Initially, the first author conducted inductive coding to identify meaning units reflecting barriers and facilitators to implementation. Independently developed codebooks by SKB and MK were compared, discussed, and consolidated into a unified coding framework, with discrepancies resolved through consensus. Codes were subsequently grouped into subthemes and mapped onto CFIR constructs to support theory-informed interpretation. Field notes and participant self-reports were analysed concurrently as complementary data sources, enabling triangulation. Selected quotations were retained to illustrate key themes and enhance trustworthiness. Although responses from open-ended questions were relatively brief, they provided sufficient depth to identify consistent patterns across participants.

The principal investigator, a native Amharic speaker and community member, had prior familiarity with participants through the screening process for intervention, which facilitated rapport but may have influenced responses. To strengthen reflexivity and analytical rigour, coding and interpretation were undertaken collaboratively within a multidisciplinary research team comprising researchers from Ethiopia, Bangladesh, Nepal, and Australia with expertise in implementation science, qualitative, public health, and behavioural research.

The quantitative fidelity data and qualitative CFIR-based findings were integrated through a mapping process. For each fidelity domain (adherence, exposure/dose, quality of delivery, participant responsiveness, and programme differentiation), we identified the CFIR constructs that participants described as facilitators or barriers to engaging with or implementing the intervention. The quantitative fidelity scores were then interpreted in light of these contextual factors to provide a comprehensive understanding of how context shaped implementation or delivery. A joint display links each fidelity domain with its associated CFIR constructs and provides an integrated interpretation (Table 3).

**Table 3:** The joint display linking each fidelity domain with its associated CFIR facilitators and barriers.

Fidelity domain	Quantitative finding	CFIR facilitators (constructs)	CFIR barriers (constructs)	Integrated interpretation
Adherence	95%	Intervention source, evidence strength and quality, adaptability	Shortened session duration for some components	High adherence reflects strong delivery of core content, although some components were delivered faster than planned.
Exposure/dose	85.71%	Adaptability, relative advantage, evidence strength and quality	Work pressure during sessions, long working hours	Attendance and session completion were good, but competing job responsibilities limited full dose exposure.
Quality of delivery	91.67%	Intervention source, evidence strength and quality, adaptability	Limited practical demonstrations (e.g., exercise, diet recipes)	Delivery quality was high, but lack of practical support may have reduced ability to apply content.
Participant responsiveness	93.75%	Intervention source, evidence strength and quality, adaptability, relative advantage (perceived relevance)	No significant barriers to engagement during intervention reported	Participants were highly engaged, attentive, and satisfied during the intervention, driven by perceived relevance and expert delivery. Contextual barriers (cost, social pressure, lack of facilities) affected post-intervention implementation, not responsiveness itself.
Programme differentiation	58.33%	Evidence strength and quality, relevance to target population	Similarity to existing programmes	The is some similarity of the programme content with national NCDs management guideline.

Source: Authors’ analysis based on study data.

## Results

### Quantitative section

#### Reach, recruitment, and retention of participants

The study successfully achieved its recruitment target, enrolling the full sample of 226 participants with equal allocation to the intervention (*n* = 113) and control (*n* = 113) arms. Retention at follow-up was high, with 207 participants (92% of those enrolled) completing endline assessments, including 105 in the intervention group and 102 in the control group.

### Fidelity of the intervention

#### Adherence

The fidelity assessment for a healthy lifestyle education intervention yielded an adherence score of 95%, indicating that all key components; including those for MetS, healthy diet, physical exercise, smoking cessation, avoidance of harmful alcohol consumption, and stress management were effectively delivered as planned. Education providers thoroughly covered the intended content, and all educational materials and resources were made available to participants. Some sessions, particularly stress management, were shorter than planned, which may have resulted in reduced exposure to this component. However, the effect of this reduced duration on participant outcomes could not be determined within the scope of this study.

#### Exposure or dose

The exposure or dose component of a healthy lifestyle education intervention fidelity yielded a score of 85.71%. On average, participants attended 75% of sessions and completed an average of three one to one sessions. This included review meetings, each lasting at least 45 min, aligning with the target period. Furthermore, participants covered at least 75% of the planned full dose for most intervention components. While there is potential for improvement in covering the full planned dose, the findings indicate a high degree of exposure to the intervention.

#### Quality of delivery

The quality of the delivery component of the intervention achieved a fidelity score of 91.67%. The educator demonstrated correct knowledge and used behaviour change techniques. The providers effectively employed behaviour change techniques in at least 75% of sessions. They successfully engaged participants and facilitated discussions on healthy lifestyles. Individualised counselling based on participants’ needs further increased the intervention’s efficacy. Additionally, the teaching materials were relevant for the prevention of MetS risks and related diseases and aligned with participants’ interests.

#### Participant responsiveness

The fidelity assessment for the participant responsiveness component of the intervention yielded a score of 93.75%. Participants showed good engagement and commitment during education sessions, actively participating in discussions. Participants correctly explained key concepts during sessions and reported high satisfaction with the one to one educational experience, indicating that personalised interactions were effective.

#### Programme differentiation

The programme differentiation component yielded a fidelity score of 58.33%. While both intervention and control conditions included similar lifestyle content aligned with national NCD guidelines, differentiation was evident in the intervention’s structured delivery, intensity, and application of behaviour change techniques. The moderate score reflects partial overlap in content domains rather than lack of distinction in delivery approach, and no evidence of contamination between groups was identified.

The open-ended questions included in the fidelity checklist indicated that relevance to the target population, engaging content, provision of reading materials, and the one to one delivery format facilitated implementation, whereas the lack of practical demonstrations of physical exercise and diet recipes limited delivery of some components.

### Qualitative section

#### Barriers and facilitators for intervention implementation

Factors that determine whether an intervention is successful or unsuccessful, as illustrated in Figure 2, included 27 identified from the 39 CFIR structures by the study. Nine of these constructs were facilitators (+) and 16 barriers (−), and 2 (complexity and cost) were present as both (±). The CFIR major domains display the emerging themes from the analysis. Table 4 provides a summary of the themes and subthemes. Illustrative participant quotes were also included.

**Figure 2:**
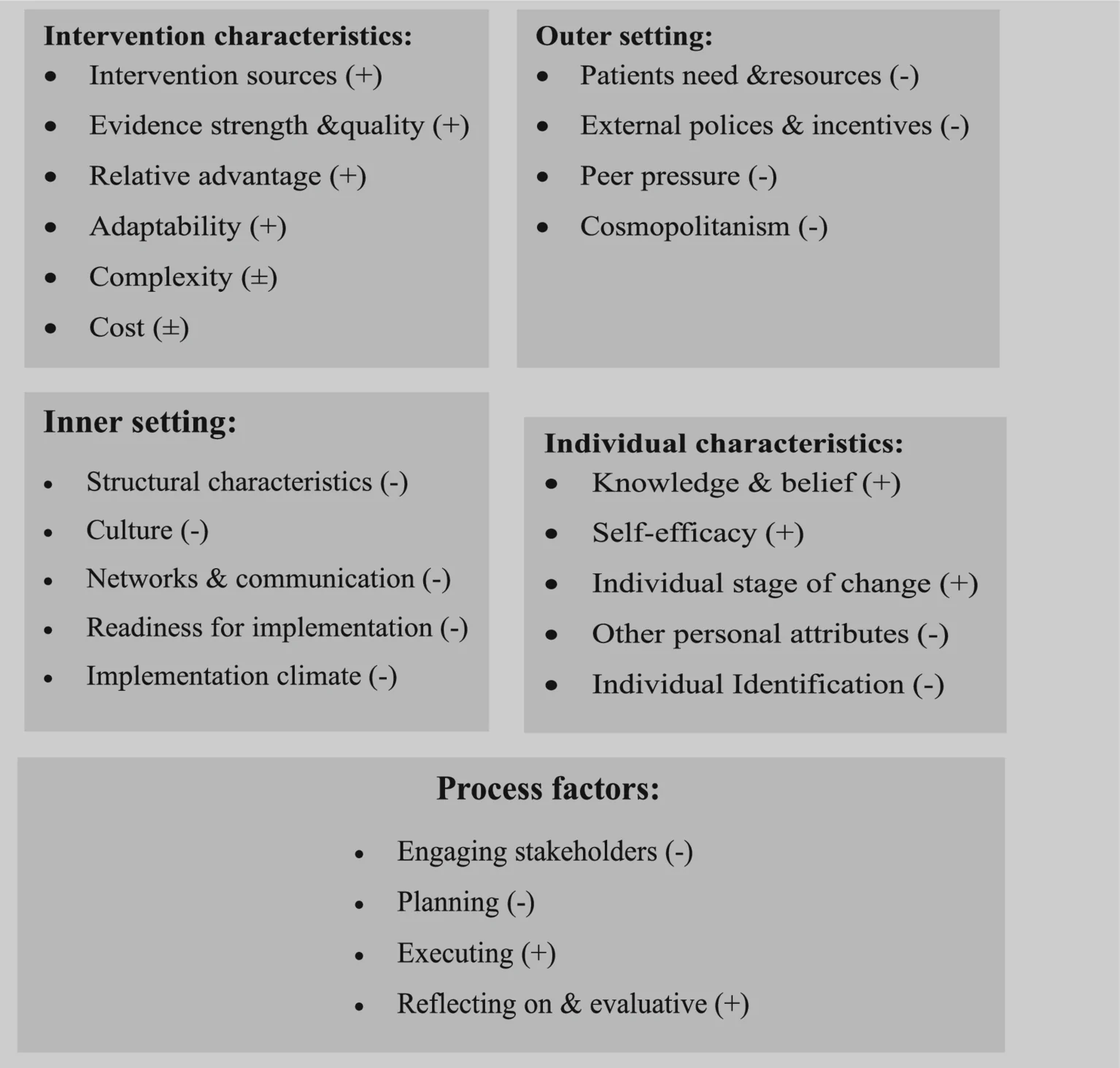
CFIR constructs and their role in implementing a workplace healthy lifestyle education intervention for metabolic syndrome risk reduction. + = facilitator, − = barrier, and ± = both. The figure is intended to organise the findings by CFIR domain only and does not indicate causal relationships, temporal sequence, or directional influence among constructs.

**Table 4:** Summary of themes and sub themes highlighting facilitators and barriers of a healthy lifestyle education intervention implementation for metabolic syndrome risk reduction.

CFIR domain and constructs	Theme	Subtheme (facilitators)	Subtheme (barriers)
Intervention characteristics			
Intervention source Evidence strength and quality Relative advantage Adaptability Complexity Cost	Expert-led and high-quality design Practical relevance and adaptability Complexity and cost for adopting healthy lifestyles	Intervention by health experts with structured, tailored education Emphasises practicalbenefits Frequent follow-up and reminders Applicability of learned behaviours in daily lives Perceived simplicity to implement Minimal direct costs	Inflated costs of healthy foods Limited facilities and environmental barriers Need for extra resources for effective implementation
Outer setting			
Patient needs and resources Cosmopolitanism Peer pressure External policies and incentives	Environmental and resource barriers Influence of societal norms External support systems	Support from online sources/experts	Limited access to healthy foods and exercise opportunities Lack of support from external health organisations and experts Cultural norms and peer pressure favouring unhealthy choices Minimal support from management, policies, and external health organisations
Inner settings			
Structural characteristics Networks and communication Culture Implementation Climate: -Tension for change -Compatibility Readiness for Implementation: -Leadership engagement -Available resources -Access to knowledge and information	Organisational culture Lack of Communication between colleagues Leadership engagement and resource limitations Challenges in implementation and accountability	Intervention aligns with personal health goals	Office environment lacks health related initiatives Organisational culture does not prioritise health or wellness initiatives Limited communication among colleagues Few incentives for adopting Healthier lifestyles Limited resources, leadership engagement, and information sharing about health among staff Limited opportunities for learning about health
Involved individual characteristics			
Knowledge and beliefs about the intervention Self-efficacy Individual stage of change Individual identification with organisation Other personal attribute	Transformation of knowledge, belief, and motivation Individual identification and support Sociodemographic characteristics	Increased confidence in making health changes post education Increased confidence and personal motivation	Initial misconceptions Limited identification with organisational health goals Lack of social support Family responsibilities Health related problems
Process factors			
Planning Engaging stakeholders Executing Reflecting on and evaluating outcomes	Participant engagement during initial planning Engagement and support from leadership Follow up and feedback mechanisms	Frequent follow up and reminders Regular assessments and feedback from researcher	Limited engagement from management and external health organisations Limited involvement during initialplanning

Source: Authors’ analysis based on study data.

### Intervention characteristics

Under this domain, four themes appeared: expert led and quality design, practical relevance, adaptability, complexity and cost for adopting healthy lifestyles. These themes embedded 6 CFIR constructs: intervention sources, evidence strength and quality, relative advantage, adaptability, complexity, and cost.

#### Expert led and quality design

The intervention’s effectiveness was significantly enhanced by the fact that it was created and conducted by trained experts (intervention source). A range of intervention components, including in person sessions with well-structured content. Both the underlying causes and preventive measures of MetS, regular text message reminders, reading materials, were addressed and interim review meetings, were implemented (evidence of strength and quality).

Participants noted that they were able to integrate the intervention into their daily routines without feeling overburdened because of the simple teaching methods that made it easy for them to understand and retain the important points (complexity). Participants thanked the expert led, well organised one to one based education for providing comprehensive explanations of MetS and the components of a healthy lifestyle (intervention sources). The teachings’ relevancy and clarity, which were closely aligned with real world scenarios, made it simpler for participants to comprehend and implement principles (evidence strength and quality).

*“The fact that the course is clear and easy to implement, that it is delivered by a professional, that it is clearly structured to address the specific problems we have, and that there is adequate supervision are among the main factors that contribute to its effectiveness” (P015).*

#### Practical relevance and adaptability

Easy adaptation was made possible by the intervention’s customisation to each person’s needs and interests (relative advantage). Participants effectively integrated new behaviours into their routines due to the training’s focus on real-world applications. Frequent check-ins, reminders, and ongoing support through calls and in-person follow-ups enhanced their motivation and commitment. Regular progress monitoring offered immediate feedback, fostering experimentation with new behaviours and promoting a sense of ownership (adaptability). For instance, participants stated that they were able to make long lasting lifestyle adjustments because of the principles’ applicability. This focus on real world application made the instruction both instructive and valuable (relative advantage).

*“The education provided is beneficial for health, but it is not complicated” (P009). “The course is tailored to my interests. Therefore, I can adapt to myself” (P005).*

#### Complexity and cost of implementation

While the intervention aimed to be simple and low direct cost (complexity and cost), participants faced significant challenges in adopting healthier behaviours. Key obstacles included the high cost of maintaining a healthy diet, limited access to sports facilities, and lack of leisure time due to demanding work schedules. Many expressed a desire for a healthier lifestyle but felt financially constrained (cost). Additionally, social, and cultural norms made unhealthy options more accessible and promoted by peers, complicating their efforts (complexity). Participants highlighted the need for increased funding for healthy diets and improved access to fitness centres (cost).

*“The lesson given is not complicated. However, it requires inputs to be implemented. The study did not include inputs needed to be implemented, such as money to buy vegetables and fruits, sports inputs, and a sports professional” (P102).*

### Outer setting

In the outer setting domain, we identified three themes: environmental and resource barriers, influence of societal norms, and external support systems. These themes align with 4 CFIR constructs: patient needs and resources, peer pressure, external policy and incentives, and cosmopolitanism.

#### Environmental and resource barriers

Political instability and limited access to healthy diets and exercise opportunities were major barriers to adopting healthier lifestyles (patient needs and resources). Transportation routes for fruits and vegetables were closing due to political unrest in the region, while the security situation and lack of local exercise facilities restricted regular physical activity (external policies and incentives). Participants found the education helpful but noted that these environmental constraints hindered their ability to apply what they learned, with many feeling that insecurity and limited resources impeded their commitment to a healthy lifestyle (patient needs and resources).

*“Due to political and security problems, the movement and the business system have been disrupted, so I have difficulty finding vegetables and fruits at the time I want them” (P102).*

#### Influence of societal norms

Societal norms significantly influenced individuals’ commitment to implementing interventions. Cultural attitudes that promote unhealthy choices, such as alcohol consumption and fatty food at social events, created pressure to conform. Participants reported that this social pressure often hindered their ability to follow healthy lifestyle recommendations. Additionally, a lack of community support and misunderstandings about healthy living made it difficult for individuals to break free from long-standing unhealthy habits (peer pressure).

*“For me, it is environmental/social influences. Because when there is a social gathering, eating fatty foods and drinking alcohol is considered a culture by society, and I am encouraged to conform to the society” (P064).*

#### External support systems

No government agencies or healthcare organisations were actively involved, and supportive policies were absent, significantly hindering the adoption of healthy lifestyles in the workplace. Participants noted major obstacles to sustaining new behaviours due to a lack of institutional support and workplace wellness programmes (external policies and incentives). The absence of engagement from external organisations limited access to valuable resources and professional guidance (cosmopolitanism), thereby reducing opportunities for support within the broader implementation environment. In the absence of such reinforcing systems and incentives (external policies and incentives), participants reported difficulty maintaining motivation and felt insufficiently supported in sustaining long-term behaviour change. Consequently, participants turned to online resources, which provided supportive advice and practical guidance on healthy lifestyle practices (cosmopolitanism).

*“I have never seen government agencies or healthcare organisations engage in such activities (P053). “Government agencies or healthcare organisations did not participate and support the implementation of the programme” (P100). “I occasionally watch articles and videos on the Internet. I also listen to professional presentations on television about how to exercise. There are also those who show us doing sports” (P102).*

### Inner setting

The evolving themes within the inner settings were organisational culture, limited communication, challenges in implementation and accountability, and management support. These themes fitted with 8 CFIR constructs: culture, structural characteristics, networks and communication, implementation climate: compatibility, readiness for implementation: available resources, implementation climate: tension for change, readiness for implementation: leadership engagement, and readiness for implementation: access to knowledge and information.

#### Workplace culture and managerial support

The workplace context created limited support for implementing the intervention’s recommendations. Participants described an organisational culture that prioritised work over health, with few health related programmes or resources and a physical environment that did not encourage healthy behaviour. The absence of onsite exercise facilities limited opportunities for physical activity during breaks or after work (culture and structural characteristics). Participants also reported limited communication about healthy lifestyles among coworkers, with few opportunities for discussion, peer encouragement, or shared problem solving (networks and communication). In addition, weak managerial support and the absence of workplace policies reduced participants’ ability to adopt and sustain healthy lifestyle changes (leadership engagement and implementation climate: compatibility). Management practices such as requiring long hours of sitting further limited opportunities for physical activity and contributed to chronic stress, reflecting (structural characteristics, implementation climate: compatibility, and readiness for implementation: access to knowledge and information).

*“Our office environment lacks health related resources and initiatives. The lack of activities and programmes has become a barrier to implementing a healthy lifestyle. For example: If our office had a gym, I could take 30 minutes off work to exercise” (P015).*

*“We have not created any ways to share information about healthy lifestyles. We only use email for work” (P082). “Neither our office culture nor management support encourage the adoption of a healthy lifestyle. We are forced to work long hours while sitting” (P015). “There is nothing to encourage a healthy lifestyle in the office environment” (P100).*

#### Challenges in implementation and accountability

When trying to put the knowledge they had learnt from the intervention into practice, participants faced a variety of difficulties (implementation climate: compatibility). Even though the instructional material was regarded as understandable and pertinent, participants complained about the absence of efficient systems for tracking their development. Their limited access to necessary resources further hampered their capacity to put their knowledge into practice (readiness for implementation: available resources). Participants also highlighted that implementing healthy behaviours is made more difficult by societal opposition to change, which often causes conflict for those who want to live healthier lives (implementation climate: tension for change). Furthermore, participants reported that work, personal duties, and the lack of accountability systems made it harder to stay committed to healthy lifestyles (implementation climate: compatibility).

*“Although the concept of implementing the given lesson was easy, there were some obstacles in putting it into practice. Among these are social life pressure, work pressure, and lack of commitment to implement it daily” (P093).*

### Involved individual characteristics

We identified two emerging themes under the domain of individual characteristics: the transformation of knowledge, beliefs, and motivation, and socio demographic factors. These themes were related to 5 CFIR constructs: knowledge and beliefs about the intervention, individual stage of change, other personal attributes, individual identification with organisation, and self-efficacy.

#### Transformation of knowledge, belief, and motivation

Many participants reported significant improvements in their knowledge, attitudes, and motivation toward healthy living because of the intervention. Initially holding misconceptions about MetS, they gained a clearer understanding of necessary lifestyle changes through in person instruction and practical resources (knowledge and beliefs about the intervention). This education not only enhanced their knowledge but also boosted their confidence to adopt healthier habits. Participants felt empowered to make positive changes, displaying greater awareness of stress management, avoiding harmful substances, making healthier food choices, and engaging in regular physical activity (self-efficacy). This indicates that the intervention effectively provided tools and encouragement for sustained behaviour change (individual stage of change).

*“I began to apply the knowledge I gained from the course with full motivation and self-confidence, and I was ready to fully implement it because I understood how important the course was for health” (P093).*

#### Socio demographic characteristics

Sociodemographic factors significantly impacted participants’ approaches to healthy living. Many struggled to find time for healthy activities due to demanding jobs, family responsibilities, and financial concerns (other personal attributes). Lasting changes were often challenging without support from family and coworkers (individual identification with the organisation).

*“Work pressure /not having enough time/, peer pressure, anxiety, security situation in the country, and cost of living are facing me as barriers to implement the given education intervention (P009).*

### Process factors

Lack of inclusive planning and follow up and feedback mechanisms were the two themes we identified under the domain of process factors. These two themes were fitted with 4 CFIR constructs: engaging stakeholders, planning, executing, and reflecting on and evaluating outcomes.

#### Lack of inclusive planning

Participants described no participation during the early planning stages of the intervention (engaging stakeholders). Their opinions were not adequately considered during the planning phase, despite the establishment of specific learning objectives and the identification of participant needs (planning). Participants stated that because they were not involved in the early planning of the intervention, specific crucial intervention components were missed during intervention development (engaging stakeholders). The lack of participant feedback limited the intervention’s potential efficacy, despite the course material being insightful and well structured (planning).

*“I was not involved in the planning of the programme. If I had been involved in the planning of the programme, I would have suggested that family members be included in the education. I would have also made sure that there was financial and material support. But since I did not have the opportunity to participate in the planning, my involvement is only in implementing the lessons I learned” (P102).*

##### Follow up and feedback mechanisms

Through a combination of in person researcher supervision, frequent reminder texts, and the use of self-reporting forms for participants to document their activities and progress, the implementation process was constantly monitored (executing). To objectively monitor changes, health evaluations, including physical measurements and laboratory tests, were conducted at the beginning and conclusion of the intervention. To sustain participant motivation, address individual problems, and tailor instructional sessions, feedback from these monitoring activities was used. This comprehensive strategy ensured the prompt identification of problems and allowed for continuous improvements to the intervention (reflecting on and evaluating outcomes).

*“I receive regular phone alerts to monitor and evaluate the implementation process. During review sessions, we discussed our implementation strategies and encountered challenges, with feedback helping improve our thoroughness and recall of important points. Physical measurements and lab tests were conducted at the beginning and end, with results reported. We also completed a form to document and report our implementation” (P088).*

#### Integrated mixed-methods findings

Table 3 presents the joint display linking each fidelity domain with its associated CFIR facilitators and barriers. It is important to note that most facilitators are derived from participant qualitative data (CFIR), while some barriers are derived from provider reports and direct observations (quantitative fidelity assessment).

High adherence (95%) and high quality of delivery (91.67%) were associated with CFIR facilitators such as expert-led design (intervention source), robust evidence base (evidence strength and quality), and flexibility to individual needs (adaptability), while shortened session duration and limited practical demonstrations were identified barriers. Exposure/dose was high (85.71%), supported by facilitators such as frequent follow-ups, reminders, and ongoing support (executing), indicating potential for improvement in full dose coverage. However, barriers such as work pressure during sessions also limited attendance. Participant responsiveness was high (93.75%) and was driven by facilitators such as perceived relevance (relative advantage), expert-led design (intervention source), and flexibility (adaptability). No barriers to engagement during the intervention were identified. Contextual barriers such as cost of healthy food, lack of sports facilities, social and peer pressure, cultural norms, and work pressure affected participants’ ability to implement recommendations after the intervention. However, this did not reduce their engagement during the intervention delivery. Programme differentiation was low (58.33%) due to similarity to existing national NCD management guideline, despite facilitators such as relevance to the target population. The detail is presented in Table 3.

## Discussion

This study contributes preliminary evidence on factors that may be considered in optimising workplace based healthy lifestyle interventions in LMIC settings and in informing future public health practice and research.

### Intervention fidelity

This process evaluation showed that the workplace healthy lifestyle education intervention was implemented with generally high fidelity and strong participant engagement, suggesting that the programme was feasible and acceptable in this Ethiopian workplace setting. These findings are consistent with literature, which emphasises that successful implementation depends not only on whether core intervention components are delivered as intended, but also on how well the intervention fits the delivery context (24). In this study, culturally responsive procedures, such as scheduling blood sample collection for fasting blood glucose and triglycerides test during fasting days, appeared to reduce participation barriers and demonstrate respect for participants’ routines. This supports the view that contextual tailoring can strengthen recruitment and retention without necessarily compromising fidelity (25, 29, 30).

The high adherence and quality-of-delivery scores indicate that the main intervention components were delivered consistently and credibly. This finding is in line with fidelity frameworks that distinguish between delivery of intervention content and the quality of interaction between provider and participant (25, 31). The expert-led, one-to-one format likely strengthened participants’ trust in the programme and increased the perceived relevance of the advice provided. Similar workplace and lifestyle intervention studies have reported that professional credibility, tailored counselling, and supportive interaction can improve engagement in health intervention programmes (32, 33). Therefore, the findings suggest that the intervention’s strength was not only the information provided, but also the way in which it was delivered.

However, fidelity was not uniform across all components. The occasional shortening of sessions and limited use of practical demonstrations indicate that interactive and skills-based activities were less consistently implemented than educational content. This pattern has also been reported in behaviour-change interventions, where informational components are often easier to deliver than experiential activities requiring time, materials, and participant practice, for instance, staff training within a national diabetes prevention programme was found to consistently cover behaviour change technique content but frequently omitted the demonstration and practice opportunities needed to build delivery skill (34), a gap that reflects the broader, still-unresolved challenge of achieving consistent fidelity across all components of complex behaviour-change interventions (35). For MetS risk reduction, this distinction is important because knowledge alone may be insufficient to achieve sustained changes in diet, physical activity, alcohol use, smoking cessation, and stress management. Future implementation should therefore protect time for demonstrations, goal-setting, problem-solving, and practice-based learning, rather than relying mainly on verbal counselling.

The dose and exposure findings further show that although attendance was good, complete dose delivery was affected by competing work demands. This is consistent with workplace health-promotion literature indicating that employee participation is shaped by organisational conditions, including workload, managerial support, scheduling flexibility, and workplace culture (36, 37), with recent reviews consistently identifying workload and scheduling conflicts as the most commonly reported organisational barriers to sustained participation in workplace-based health interventions (36). Thus, reach and attendance should not be interpreted as sufficient evidence of implementation success. Even motivated participants may receive a reduced intervention dose if workplace systems do not protect time for participation. For scale-up, employer commitment, supervisor support, and integration of intervention activities into routine work schedules will be necessary to maintain fidelity.

Programme differentiation was lower than other fidelity domains because some intervention content overlapped with existing noncommunicable disease prevention guidance. This should not be interpreted as a weakness, since the intervention was designed to operationalise evidence-based lifestyle recommendations rather than provide entirely novel advice. In this context, the programme’s added value was its tailored, expert-delivered, and interactive approach. Fidelity literature suggests that differentiation is most relevant when an intervention must be clearly distinguished from usual care, with recent work emphasising that fidelity assessment should centre on whether an intervention’s essential components are present and delivered with sufficient strength, rather than on whether its content is entirely novel (38). Interventions explicitly built to operationalise existing evidence-based guidelines, rather than introduce novel content, may reasonably score lower on this domain without this reflecting a design or delivery flaw (39). Where overlap is intentional, the key issue is whether the programme improves delivery, uptake, and contextual relevance.

### Contextual factors influencing lifestyle intervention implementation

This study also investigated the facilitators and barriers (assessed through participant perceptions) to putting the intervention into practice using the CFIR. The findings showed that implementation was shaped by interacting factors across intervention, individual, organisational, sociocultural, and structural domains. Although participants perceived the intervention as relevant and credible, their ability to apply recommendations depended on the realities of their work, household, social, and economic environments. This supports the Consolidated Framework for Implementation Research, which emphasises that implementation outcomes are influenced by intervention characteristics, inner setting, outer setting, individual characteristics, and implementation processes (25, 29). The findings also align with socioecological perspectives, which argue that health behaviour is not produced by individual motivation alone but by the interaction between people and their environments (40, 41).

Intervention characteristics, particularly expert delivery, clarity, and adaptability, were important facilitators. Participants valued the opportunity to receive tailored guidance that could be adjusted to their daily routines, occupational responsibilities, and household obligations. This is consistent with recent evidence showing that adaptable interventions are more likely to be acceptable and sustainable because they allow core intervention functions to be maintained while delivery is modified to fit local needs (42, 43). In the present study, adaptability appeared to support initial engagement and confidence. However, it did not fully overcome broader constraints such as food cost, limited exercise facilities, insecurity, and workload. This suggests that adaptable counselling is necessary but insufficient when structural barriers restrict participants’ capacity to act.

Economic barriers were particularly important in relation to healthy eating and physical activity. Participants reported that recommended dietary changes could be difficult to implement because of food cost and availability. This finding is consistent with global evidence showing that healthier diets are often less affordable for low- and middle-income populations and that food environments strongly influence dietary behaviour (44, 45). Similarly, opportunities for physical activity depend not only on personal motivation but also on time, safety, facilities, and supportive environments (46, 47). Therefore, limited behaviour change should not be interpreted simply as poor adherence. Rather, it reflects the difficulty of implementing lifestyle advice under constrained economic and environmental conditions.

Sociocultural norms also influenced implementation. Participants described how alcohol consumption, preference for fatty foods, and expectations during social gatherings conflicted with the intervention’s recommendations. These findings are consistent with literature showing that diet and alcohol-related behaviours are socially embedded and shaped by family roles, peer expectations, hospitality practices, and community identity (48, 49). This is especially relevant in lifestyle interventions because recommendations that appear simple at the individual level may become difficult to practise in social contexts where shared meals and celebrations carry cultural meaning. The findings therefore suggest that future interventions should not frame cultural practices as barriers to be rejected, but should work with participants to identify acceptable modifications, involve family members where appropriate, and promote healthier versions of customary practices.

The workplace environment was another central influence on implementation. Limited organisational support, lack of wellness structures, sedentary work routines, competing job demands, and weak peer-support mechanisms constrained participants’ ability to integrate lifestyle changes into daily routines. This finding is consistent with workplace health-promotion evidence showing that leadership support, organisational culture, policy alignment, and environmental resources are major determinants of programme participation and sustainability (50, 51). It also helps explain why high individual responsiveness during intervention delivery may not lead automatically to sustained behaviour change. Without supportive workplace systems, responsibility for change remains largely with individuals, even when many barriers are organisational.

Participants’ increased knowledge, motivation, and confidence indicate that the intervention strengthened important individual-level determinants of behaviour change. This is consistent with behaviour-change literature showing that risk awareness, self-efficacy, action planning, feedback, and self-monitoring can support lifestyle modification (52, 53). However, the qualitative findings show that these individual gains were moderated by workload, family responsibilities, financial constraints, health conditions, and limited environmental support. The study therefore supports an important interpretation: education can initiate change, but sustained implementation requires enabling conditions. For MetS risk reduction, this means that counselling should be combined with practical strategies such as affordable meal planning, home-based physical activity options, peer support, and follow-up mechanisms.

Family involvement emerged as a potentially important strategy for improving implementation. Participants suggested that including family members could strengthen dietary change, physical activity, and adherence to recommendations. This is consistent with evidence from chronic disease and diabetes self-management research showing that family support can improve behaviour change by influencing food purchasing, meal preparation, encouragement, accountability, and shared routines (54–56). Given that diet and physical activity are often household-level behaviours, future workplace interventions may benefit from adding family-oriented education materials, household goal-setting, or optional family sessions.

Overall, this implementation process evaluation study demonstrated that the intervention was delivered with strong fidelity, but implementability was limited by workplace, economic, sociocultural, and structural constraints. The findings highlight the need for multilevel, context-responsive implementation strategies that include workplace support, peer engagement, culturally tailored counselling, family involvement, and low-cost diet and physical activity options to enhance fidelity, implementability, and equity in MetS risk reduction.

### Implications for policy and practice

This study indicates that a theory-informed, expert-led, individually delivered workplace lifestyle education intervention can be implemented with high fidelity and strong participant engagement among office workers, reinforcing the value of professional workplaces as promising settings for NCD and metabolic risk prevention. At the same time, the findings suggest that routine implementation will require careful adaptation to workforce constraints, limited institutional support, and variable resource availability.

A major implication is that the intervention may be amenable to task-sharing or task-shifting within workplace and primary care systems. Core activities such as lifestyle education, goal setting, reminders, basic risk monitoring, follow-up support, and referral facilitation could potentially be delegated to trained nurses, community health workers, occupational health personnel, or workplace peer champions. However, such adaptation would need to be supported by clear protocols, competency-based training, supervision, fidelity monitoring, and referral pathways to preserve intervention quality and continuity.

This is especially relevant given the barriers identified in the study, including limited resources, weak organisational support, and minimal external healthcare involvement. Rather than depending solely on specialist-led delivery, future implementation models in Ethiopian workplaces may benefit from shared-care approaches that distribute responsibilities across available personnel while maintaining structured oversight. Emerging evidence suggests that task-sharing and task-shifting can support chronic disease and multimorbidity management in resource-constrained settings (57, 58), although their feasibility and fidelity should be tested in Ethiopian workplace contexts.

Policy efforts should also focus on embedding workplace health promotion within organisational systems. This could include incorporating lifestyle support into human resource policies, protecting time for health activities, strengthening workplace wellness infrastructure, and fostering partnerships between employers, occupational health services, and public health institutions. Such measures would help shift workplace interventions from isolated initiatives to routine organisational practice.

At the broader policy level, structural support is needed to reduce barriers to healthy living. These may include incentives for healthier food options, collaboration with local authorities to promote supportive social norms, and government action such as subsidies for healthy foods, urban design that supports active transport, and stronger regulation of alcohol and unhealthy product marketing. Together, these strategies may inform future efforts to improve the scalability, equity, and sustainability of workplace-based NCD prevention in Ethiopia and comparable low- and middle-income settings, although these outcomes were not directly assessed in the present study. Future research should examine implementation costs, cost-effectiveness, long-term maintenance, and conditions for scale-up.

### Strengths and limitations

A key strength of this study is its comprehensive evaluation of intervention fidelity and implementation using both the PIF and CFIR, enabling systematic examination of multilevel facilitators and barriers within a real-world LMIC workplace setting. The use of mixed methods and a joint display further strengthened analytic integration, allowing quantitative fidelity scores to be interpreted alongside contextual and organisational influences. However, several limitations should be acknowledged.

First, fidelity assessment was conducted with only one of the three intervention providers. Although the selected provider delivered the largest proportion of sessions and thus represented a substantial segment of delivery, the absence of fidelity data from the other two providers limits the ability to assess inter-provider variability and may reduce the generalisability of fidelity findings across all implementers. Second, direct observations were conducted by the principal investigator, who was also involved in the study. This may have introduced observer bias, whether conscious or unconscious, in scoring fidelity domains. Although structured checklists were used to standardise observations, fidelity ratings were not independently verified or double-coded, which limits the ability to fully rule out measurement bias. Third, educator self-reports contributed to fidelity assessment and may have been subject to self-report and social desirability bias, potentially inflating adherence, and quality ratings. The absence of independent verification of these self-reported components further increases the possibility of overestimation.

Fourth, qualitative data were collected only from intervention completers. Excluding non-completers may have resulted in underrepresentation of negative experiences or disengagement-related barriers, thereby potentially biasing the findings toward more favourable perceptions of implementation. Fifth, the qualitative sample was small (13 participants purposively selected from 105), and data were gathered through written open-ended responses rather than in-depth interviews. While this approach facilitated participation within a workplace context, it may have limited the depth, nuance, and opportunity for probing that richer qualitative methods could provide. Sixth, the perspectives of employers, managers, and intervention providers were not included in the qualitative component. As a result, organisational-level determinants, such as leadership engagement, resource allocation, and institutional readiness, may not have been fully captured, limiting a more comprehensive understanding of inner-setting influences. Seventh, as interviews were conducted in the local language and translated into English, there is potential for subtle translation inaccuracies or loss of cultural nuance despite efforts to preserve semantic meaning. Such issues may have affected interpretation of qualitative findings.

Finally, the study was conducted among bank employees in Bahir Dar, which may limit transferability to other occupational groups, regions, or broader community populations. Workplace structures, job demands, and organisational cultures differ across settings; therefore, caution is warranted when extrapolating these findings beyond similar urban office-based environments in comparable contexts.

## Conclusion

This process evaluation suggests that the workplace lifestyle intervention was implemented with generally high fidelity across key domains, including adherence, quality of delivery, exposure, and participant responsiveness. The findings indicate that the intervention was delivered largely as intended; however, this conclusion should be interpreted with caution due to the reliance on self-reported and potentially non-independent measures. The absence of triangulation or external verification may introduce bias, and therefore the extent of fidelity cannot be definitively confirmed. Participant-reported facilitators included perceived relevance, engaging and personalised delivery, and the provision of written materials. Reported barriers included work-related time pressures, limited practical demonstrations, and environmental constraints that influenced participants’ ability to fully engage with or apply certain components.

Programme differentiation was comparatively low, reflecting partial overlap with existing national NCD management guidance, although the intervention was distinct in its individualised, one to one, and workplace-based delivery approach. Overall, the implementation was shaped by both delivery-level factors and broader contextual conditions.

These findings provide process-level evidence to inform refinement of the intervention and its delivery model. Issues related to national policy integration, scalability, long-term sustainability, equity, and cost-effectiveness were not directly assessed in this evaluation and should be addressed in future research.

## Data Availability

The original contributions presented in the study are included in the article/Supplementary material, further inquiries can be directed to the corresponding author.
