# Correction: Implementation of a workplace healthy lifestyle education intervention for metabolic syndrome risk reduction in Ethiopia: a mixed-methods process evaluation

**DOI:** 10.3389/fpubh.2026.2025861

**Published:** 2026-10-21

**Authors:** Sitotaw Kerie Bogale, Haribondhu Sarma, Uday Narayan Yadav, Matthew Kelly

**Affiliations:** 1 National Centre for Epidemiology and Population Health, The Australian National University, Canberra, ACT, Australia; 2 College of Medicine and Health Sciences, Bahir Dar University, Bahir Dar, Ethiopia; 3 International Centre for Future Health Systems, University of New South Wales, Sydney, NSW, Australia; 4 The Poche Centre for Indigenous Health, Faculty of Medicine and Health, The University of Sydney, Sydney, NSW, Australia

**Keywords:** Ethiopia, fidelity, implementation, lifestyle intervention, metabolic syndrome, office workers, risk factors, workplace

Affiliation College of Medicine and Health Sciences, Bahir Dar University, Bahir Dar, Ethiopia was omitted from the paper. This affiliation has now been added for author Sitotaw Kerie Bogale.

Affiliation International Centre for Future Health Systems, University of New South Wales, Sydney, NSW, Australia was erroneously assigned to Sitotaw Kerie Bogale. This affiliation has now been removed for author Sitotaw Kerie Bogale.

The original version of this article has been updated.

