# Supplementary material for: Implementation of a workplace healthy lifestyle education intervention for metabolic syndrome risk reduction in Ethiopia: a mixed-methods process evaluation
Source: Front Public Health. 2026 Oct 7;14:1893222. doi: 10.3389/fpubh.2026.1893222 (PMC13683782; doi:10.3389/fpubh.2026.1893222)
Supplement: Supplementary file 1 [file Data_Sheet_1.PDF]

Fidelity Checklist for One-on-One based Education Interventions promoting a healthy lifestyle to reduce metabolic risk factors for Office Workers in Ethiopia.

S.no	Domains	Responses	Answer
**1**	**Adherence**		
	Were all the key intervention components (healthy diet, physical exercise, quitting smoking, avoiding harmful alcohol consumption, and stress management) delivered as intended?	Yes, all components were delivered = 4 points<br>Some components were delivered = 2 points<br>No, none of the components were delivered = 0 points	
	Was the intended content covered for each intervention component?	Yes, all contents were covered = 4 points<br>Some contents were covered= 2 points<br>No, none of the contents were delivered = 0 points	
	Were the scheduled education sessions delivered one-on-one as planned?	Yes, all education sessions delivered as planned= 4 points<br>Some education sessions delivered as planned = 2 points<br>No, none of the education sessions delivered as planned = 0 points	
	Was the intended session duration and frequency adhering to the protocol?	Yes, all session duration and frequency adhered to the protocol= 4 points<br>Some session duration and frequency adhered to the protocol= 2 points<br>No, none of the session duration and frequency adhered to the protocol= 0 points	
	Were the educational materials and resources provided to participants as planned?	Yes, all materials were provided = 4 points<br>Some materials were provided = 2 point<br>No, no materials were provided = 0 points	
		**Total score**	**/ 20%**

**2**	**Exposure or Dose**		
	Did participants attend the scheduled education sessions as intended?	Yes, participants participated at all scheduled sessions = 4 points<br>Participants attended most (at least 75%) of the planned sessions = 3 points<br>Participants participated at some (50-74%) of the scheduled sessions = 2 points<br>Participants attended few (less than 50%) of the planned sessions = 0 points	
	What was the average number of one-on-one sessions completed by participants?	4 or more sessions = 4 points<br>2-3 sessions = 2 points<br>1 session or less = 0 points	
	What was the average duration of the one-on-one sessions?	45 minutes or more = 2 points<br>30-44 minutes = 1 point<br>Less than 30 minutes = 0 points	
	Did participants receive the full planned dose of each intervention component?	Yes, participants received the full planned dose of all components = 4 points<br>Participants received the full planned dose of most (at least 75%) components = 3 points<br>Participants received the full planned dose of some (50-74%) components = 2 points<br>Participants received the full planned dose of few (less than 50%) components = 0 points	
		**Total score**	**/ 14%**
**3**	**Quality of Delivery**		
	How would you rate the competence and skill of the educators/facilitators in delivering the content?	Educator demonstrated correct knowledge and used behaviour change techniques = 4 points	

		Moderately demonstrated correct knowledge and used behaviour change techniques = 2 points<br>Not demonstrated correct knowledge and used behaviour change techniques = 0 points	
	Did the educators use the appropriate behavior change techniques (e.g., goal-setting, self-monitoring, problem-solving) as intended?	Yes, the educators used all the appropriate behavior change techniques as intended = 4 points<br>The educators used most (at least 75%) of the appropriate behavior change techniques as intended = 3 points<br>The educators used some (50-74%) of the appropriate behavior change techniques as intended = 2 points<br>The educators used few (less than 50%) of the appropriate behavior change techniques as intended = 0 points	
	Were the educators able to engage participants and facilitate interactive discussions during the sessions?	Yes, the educators were successfully engaged participants in ≥75% of sessions and facilitating discussions = 4 points<br>The educators were moderately effective at engaging participants and facilitating discussions = 2 points<br>The educators were not effective at engaging participants or facilitating discussions = 0 points	
	Did the educators provide personalized guidance and support to participants as needed?	Yes, the educators provided high-quality personalized guidance and support = 4 points<br>The educators provided some personalized guidance and support = 2 points<br>The educators did not provide personalized guidance and support = 0 points	

	To what extent were the educational materials and activities clear, relevant, and engaging for participants?	Very clear, relevant, and engaging = 2 points<br>Somewhat clear, relevant, and engaging = 1 point<br>Not clear, relevant, or engaging = 0 points	
			**/18%**
**4**	**Participant Responsiveness**		
	Were participants engaged and attentive during the education sessions?	Yes, participants were highly engaged and attentive = 4 points<br>Participants were somewhat engaged and attentive = 2 points<br>Participants were not engaged or attentive = 0 points	
	Did participants actively participate in discussions and complete the planned activities?	Yes, participants actively participated in discussions and completed all activities = 4 points<br>Participants participated in some discussions and completed most activities = 2 points<br>Participants did not participate in discussions or complete activities = 0 points	
	Did participants understand the key concepts and skills taught in the sessions?	Yes, participants correctly explained key concepts during sessions = 4 points<br>Participants demonstrated a moderate participant correctly explained key concepts during sessions = 2 points<br>Participants did not correctly explain key concepts during sessions = 0 points	
	Did participants report satisfaction with the one-on-one educational experience?	Yes, participants reported high satisfaction = 4 points<br>Participants reported moderate satisfaction = 2 points<br>Participants reported low or no satisfaction = 0 points	
		**Total score**	**/16%**
**5**	**Program Differentiation**		

	Were there clear differences between the one-on-one education intervention and any usual care or other comparison conditions?	Yes, there were clear and substantial differences = 4 points<br>There were some differences, but they were not very distinct = 2 points<br>There were no clear differences between the intervention and comparison conditions = 0 points	
	Were the specific intervention components (healthy diet, physical exercise, smoking cessation, alcohol reduction, stress management) distinct from each other and any other programs or services available to participants?	Yes, the components were highly distinct and differentiated = 4 points<br>The components had some overlap with other programs but were still distinct = 2 points<br>The components were not distinct from other available programs = 0 points	
	Were there mechanisms in place to monitor and ensure that the one-on-one education intervention was implemented as a unique and differentiated program?	Yes, there were robust monitoring mechanisms in place = 4 points<br>There were some monitoring mechanisms, but they were not comprehensive = 2 points<br>There were no mechanisms in place to monitor program differentiation = 0 points	
		**Total score**	**/12%**

1. What characteristics of the intervention itself facilitated its delivery? (e.g., relevance to the target population, ease of implementation, engaging content, providing reading material during education, etc.)-- -- --.
2. What characteristics of the intervention itself presented barriers to its delivery? (e.g., complexity, lack of cultural relevance, perceived irrelevance, etc.)-- -- ---.
