# Supplementary material for: Implementation of a workplace healthy lifestyle education intervention for metabolic syndrome risk reduction in Ethiopia: a mixed-methods process evaluation
Source: Front Public Health. 2026 Oct 7;14:1893222. doi: 10.3389/fpubh.2026.1893222 (PMC13683782; doi:10.3389/fpubh.2026.1893222)
Supplement: Supplementary file 2 [file Data_Sheet_2.PDF]

Open-ended questions to evaluate the implementation process of a healthy lifestyle education intervention to reduce metabolic syndrome risk factors among office workers in Ethiopia, based on the CFIR domains:

Intervention Characteristics:

1. Can you describe the key components of the education intervention, such as the topics covered and the teaching methods used?
2. How complex or simple was the intervention to implement in terms of the resources required and the level of coordination needed?
3. To what extent could you adapt or tailor the intervention to your specific needs and preferences?
4. What did you know about the evidence or rationale behind the effectiveness of this particular intervention?

Outer Setting:

1. What broader factors within Ethiopia, such as policies, regulations, or social norms, influenced your experience with the intervention?
2. Were there any external support or barriers, such as workplace wellness programs or health insurance coverage, weather conditions, etc…that affected the implementation of the intervention?
3. How engaged and supportive were external stakeholders, such as government agencies or healthcare organizations, towards implementing the intervention?

Inner Setting:

1. Can you describe the organizational characteristics of your office environment, including the available resources and existing health-related initiatives?
2. What was the organizational culture and level of management support towards implementing the intervention?
3. How effective were the communication channels and information-sharing mechanisms among your colleagues in disseminating and adopting the intervention?
4. Were there any existing programs or initiatives related to healthy lifestyle promotion that complemented or competed with the implementation of the intervention?

Characteristics of Individuals:

1. What were your knowledge, attitudes, and beliefs regarding the importance of a healthy lifestyle and the risk factors for metabolic syndrome?
2. How motivated and confident were you in your ability to adopt and maintain the healthy lifestyle behaviors promoted by the intervention?
3. Were there any personal or cultural factors that influenced your interest to the intervention?

Process:

1. Can you describe your involvement or the involvement of your colleagues in the planning and adaptation of the intervention?
2. What strategies were used to engage and educate you about the intervention, and how effective were they?
3. What mechanisms were in place to monitor and evaluate the implementation process, and how were the findings used to improve the intervention?

Any other feedback:

Thanks!!!
